# Exploring treatment outcomes in Stage II-III rectal cancer patients undergoing neoadjuvant therapy at a tertiary care center in Pakistan: a comprehensive analysis of pathological outcomes

**DOI:** 10.1186/s12885-024-12241-6

**Published:** 2024-04-16

**Authors:** Misbah Younus Soomro, Saqib Raza Khan, Hafiz Muhammad, Sujjawal Ahmad, Nawazish Zehra, Insia Ali, Mirza Rameez Samar, Arif Hameed, Munira Moosajee, Yasmin Abdul Rashid

**Affiliations:** 1 Aga Khan University Hospital, Karachi, Pakistan; 2 Aga Khan University Centre for Regenerative Medicine and Stem Cell Research, Karachi, Pakistan; 3 Department of Medical Oncology, Section of Oncology, Aga Khan University Hospital, Karachi, Pakistan

**Keywords:** Rectal cancer, Pathological response, Chemotherapy, Radiation therapy, Outcomes

## Abstract

**Background:**

Rectal cancer treatment has transformed in recent years, with neoadjuvant treatment (NT) and total neoadjuvant treatment (TNT) aiming to enhance pathological responses. This pioneering study in our country delves into rectal cancer management, offering crucial insights by examining pathological outcomes in patients treated with the NT and TNT approach, shaping the evolving landscape.

**Methods:**

In this retrospective-cohort study spanning January 2017 to December 2022 at a tertiary care hospital in Pakistan, ethical approval was obtained to examine outcomes of two treatments. Patients were divided into TNT (chemoradiation and pre-surgery 5 FU-based chemotherapy) and NT (chemoradiation, surgery, and subsequent 5 FU-based chemotherapy). The primary end-point was response rates—no response, pathological complete response (pCR), near complete response (near CR), and partial response (PR). The Chi-Square Test for Independence assessed the association between treatment response and type (TNT or NT). Data analysis used STATA MP 64; significance was set at *p* < 0.05 for all two-tailed tests.

**Results:**

We analyzed 77 patients, 60 underwent standard neoadjuvant chemoradiation, and 17 followed the total neoadjuvant approach. Predominantly male, most were > 65 with ECOG 0–1. The TNT group showed higher response rates (76% vs 62%, *p* = 0.039), with 40.38% achieving pCR. In the overall population, pCR and near-CR were similar (27.2% vs 26%), while PR were 14%. Treatment characteristics correlated significantly with chemotherapy type, concurrent chemoradiation, LVI, PNI, and T, N, M staging (*p* < 0.05). Median overall survival was not reached, and mean survival was 89.1 months (CI: 95.0 to 83.3). Side effects varied, with notable differences in neuropathy, diarrhea, oral mucositis, and thrombocytopenia between NT and TNT groups.

**Conclusion:**

Our study adds to evidence favoring neoadjuvant approaches in managing rectal cancer in pakistan. Demonstrating a favorable pcr rate, ongoing research with extended follow-up is essential, given the dynamic landscape of rectal cancer treatment for improved patient outcomes.

## Introduction

The landscape of rectal cancer treatment has evolved significantly in recent years, reflecting a dynamic shift in the care paradigm. Colorectal cancers, constituting the third most prevalent cancer globally, contribute to approximately 10% of newly diagnosed cancer cases worldwide [1]. Strikingly, in Pakistan, recent data from the National Cancer Registry positions colorectal cancer as the second most common cancer, underscoring its considerable impact on the local population [2].

Rectal cancers encompass malignancies arising from the rectum. The American Joint Committee on Cancer (AJCC) TNM staging delineates Stage II rectal cancers as node-negative, while Stage III includes node-positive disease without distant metastasis. The latter category often necessitates a comprehensive treatment approach involving chemotherapy, radiation, and surgical resection [1, 2].

In our population, colorectal cancers account for 4.8% of all new cancer cases, with rectal Carcinoma constituting 1.8% of these cases. The mortality rate for rectal cancer in Pakistan stands at 1.5% of all cancer-related deaths [2]. Traditionally, the predominant treatment approach has centered around neoadjuvant therapy, involving concurrent chemoradiation therapy followed by surgery and adjuvant chemotherapy—a total perioperative treatment spanning six months. A retrospective review 2016 reported a pathological complete response (pCR) rate of 12% for patients treated with this neoadjuvant approach [3]. However, a subsequent study in 2021, encompassing data from 2007 to 2014, indicated a notable improvement with a pCR rate of 52% and an acceptable toxicity profile [4].

In recent years, there has been a paradigm shift towards total neoadjuvant treatment (TNT) as the new standard for locally advanced rectal cancer. This innovative approach incorporates radiation therapy, either with a short course of radiation therapy (RT) or a long course of RT concurrent with chemotherapy and systemic chemotherapy preceding surgery after completing systemic treatment [5]. Excitingly, the exploration of neoadjuvant immunotherapy has revealed promising results in patients with microsatellite-high (MSI-H) locally advanced rectal tumors, showcasing durable responses [5, 6].

Chemotherapeutic agents’ integral to concurrent chemoradiation and perioperative settings include 5-FU-based systemic chemotherapy. Both oral capecitabine and intravenous 5-fluorouracil are deemed valuable options, demonstrating no discernible difference in outcomes [5]. The principal toxicities associated with these agents encompass diarrhea, neutropenia, and oral mucositis [7].

However, despite the burgeoning literature on rectal cancer treatment globally, none of the published studies from this region have specifically scrutinized patients undergoing neoadjuvant and total neoadjuvant treatment. This lacuna in the literature prompted the initiation of our study, which aims to evaluate patients treated with neoadjuvant and total neoadjuvant approaches comprehensively. Hence, This study is going to evaluate how patients in our part of the world response to neoadjuvant and total neoadjuvant approach in real life as compared to the clinical trial results. Our study will delve into the toxicity profile, and the subsequent impact on surgical outcomes within our patient population. This pioneering effort represents the first of its kind in our country, providing invaluable insights into the evolving landscape of rectal cancer management [1-5, 7].

## Methods, treatment and statistical analysis

We employed a retrospective cohort design for our study, where we reviewed patients' medical records from January 2017 to December 2022. Approval was obtained from the Ethical Review Committee (ERC), and then the study was conducted at our tertiary care hospital in the Department of Medical Oncology in Karachi, Pakistan.

We utilized a non-probability purposive sampling technique for patient selection. Patients with European Cooperative Oncology Group (ECOG) performance status of 0-II [(ECOG-0: Fully active, ECOG-I: Restricted in physically strenuous activity but ambulatory and able to carry out work of a light or sedentary nature, ECOG-II: Ambulatory and capable of all self-care but unable to carry out any work activities; up and about more than 50% of waking hours)], stage II/III rectal adenocarcinomas, according to the AJCC 8 classification and who had completed their treatment at our Centre and received neoadjuvant or total neoadjuvant treatment followed by surgery, were included. Patients who had undergone upfront resection and those who developed metastatic disease during treatment were excluded. Out of 89, a total of 77 patients who fulfilled our study inclusion criteria were selected for study analysis. We reviewed all patients' baseline characteristics and their medical records for information such as age, ECOG status, comorbidities, and tumor characteristics, including clinical stage, pathological TNM stage, LVI, and PNI (Tables 1 and 2).

**Table 1:** Baseline patient and disease characteristics of patients receiving treatment

Patient and disease characteristics		Standard therapy (NT), *n* = 60, (%)	TNT, *n* = 17, (%)
**Age in year**	**< 65**	14 (23.3)	16 (94.1)
	**≥ 65**	46 (76.6)	01 (5.9)
**Gender**	**M**	40(66.67)	08 (47.1)
	**F**	20(33.33)	09 (52.94)
**ECOG performance status scale**	**0**	15(25)	01 (5.9)
	**1**	41(68)	13 (76.5)
	**2**	04(6.67)	03 (17.64)
**Clinical stage**	**1**	0 (00)	0 (00)
	**2**	09 (15)	0 (00)
	**3**	51 (85)	17 (100)
	**4**	0(00)	0 (0.00)
**Addiction**	**Smoker**	18 (30.00)	05 (29.4)
	**Alcoholic**	02(3.33)	01(5.8)
	**Unknown**	40(66.66)	11 (64.7)
**Comorbidities**	**Hypertension**	15 (25.00)	03 (17.66)
	**Diabetes Mellitus**	05 (8.33)	02 (11.76)
	**Ischemic Heart Disease**	01 (1.66)	01 (5.88)
	**Hypo/Hyperthyroidism**	0	01 (5.88)
	**Others (Tuberculosis, Asthma, Polio, Ulcerative Colitis)**	0	0
	**Unknown**	39 (65)	10 (58.82)

*ECOG* Eastern cooperative oncology group performance status, *TNT* Total neoadjuvant treatment, *NT* Neoadjuvant

**Table 2:** Treatment characteristics

	**Treatment characteristics**			
**Characteristic**		**Standard therapy (NT), *n* = 60 (%)**	**TNT, *n* = 17 (%)**	**Significance**
**Chemotherapy regimen**	**CAPEOX**	52 (86.6)	12(70.5)	0.043*
	**m-FOLFOX6**	08 (13.3)	05(29.4)	
**Surgery**	**Abdominoperineal Resection (APR)**	29 (13.3)	11(64.7)	0.70
	**Low Anterior Resection (LAR)**	31(51.6)	06(35.2)	
**Concurrent chemo radiotherapy (CCRT)**	**Yes**	60 (100)	08(47)	0.047*
	**No**	00	09(53)	
**Radiation Therapy (RT)**	**Short**	00	09(53)	0.08
	**Long**	00	08(47)	
**Histological grade**	**Well-differentiated**	47(78.00)	07 (41.20)	0.90
	**Moderately differentiated**	10(17.00)	09 (53.00)	
	**Poorly differentiated**	03(5.00)	01 (5.80)	
**Circumferential Resection Margin (CRM)**	**Positive**	03 (05)	01 (5.9)	0.80
	**Negative**	43 (71.66)	14 (82.4)	
	**Closed**	14 (23.33)	02 (11.76)	
**Perineural Invasion (PNI)**	**Present**	10 (16.66)	03 (17.64)	0.049*
	**Absent**	50 (83.33)	14 (82.35)	
**Lymph Vascular Invasion (LVI)**	**Present**	8(13.33)	02(11.76)	0.047*
	**Absent**	44(73.33)	12 (70.58)	
	**Indeterminate**	8(13.33)	03(17.76)	
**T stage**	**T0**	12 (20)	09 (52.9)	0.01*
	**T1**	06 (10)	01 (5.8	
	**T2**	22 (36.6)	02 (11.7)	
	**T3**	17 (28.33)	04 (23.5)	
	**T4**	03 (05)	01 (5.8)	
**N stage**	**N0**	25(41.66)	12 (70.58)	0.03*
	**N1**	20 (33.33)	03 (05.00)	
	**N2**	15 (25.00)	02 (11.76)	
**M stage**	**M0**	52 (86.6)	15 (88.2)	0.01*
	**M1**	08 (13.3)	02 (11.7)	

*Statistically significant results

Patients were categorized into two groups: those who underwent TNT (comprising patients who received chemoradiation and 5FU-based chemotherapy cycles before definitive surgery) and those who underwent NT (which involved chemoradiation, followed by surgery and subsequent chemotherapy with a 5 FU-based regimen). All patients in the NT group received chemoradiation therapy with capecitabine 825 mg/m^2^ PO twice daily throughout radiation therapy (28–30-day course) followed by surgery. This is followed by a continuation of systemic chemotherapy with either a combination of capecitabine 1000 mg/ m^2^ from day 1 to day 14 and oxaliplatin 130 mg/ m^2^ on day 1 of every 21-day cycle (CAPEOX) or oxaliplatin 85 mg/m^2^ IV on day 1, leucovorin 400 mg/m^2^ IV on day1, 5FU 400 mg/m^2^ IV bolus on day one followed by 2400 mg/m^2^ IV for 46–48 h’ continuous infusion (mFOLFOX6), every two weekly (6 months of perioperative treatment). All patients in the TNT group received radiation therapy (either short course RT or long course concurrent with capecitabine) and 5 FU-based systemic chemotherapy (CAPEOX or mFOLFOX6) and then definitive surgery. All patients were followed closely after completion of treatment with history, physical examination, and periodic systemic scans.

The primary endpoint was response rates, which were further categorized into no response, pathological complete response (pCR), near complete response (near CR) and partial response (PR). We used the *Modified Ryan Scheme* for tumor regression scoring in rectal cancer to determine the Response [8]. We defined pCR as the microscopical absence of tumor cells in tissue samples removed after surgery. We defined near-complete responses as single or rare small-group cancer cells in the tissue sample removed after surgery. We defined partial response as residual cancer with evident tumor regression but more than single cells or rare small groups of cancer cells. We defined no response as extensive residual cancer with no apparent tumor regression (Table 3). We also reviewed high-risk factors and pathological outcomes after surgery.

**Table 3:** Modified Ryan scheme for tumor regression scoring in rectal cancer treated preoperatively

S.No	Description	Regression score
1	No viable cancer cells	0 (complete response)
2	Single cells or rare small groups of cancer cells	1 (near-complete response)
3	Residual cancer with evident tumor regression	2 (partial response)
4	Extensive residual cancer with no apparent tumor regression	3 (no response)

For our study, we selected patients who had undergone either APR (abdominal perineal resection, which includes the resection of the sigmoid colon, rectum, and anus, with the construction of a permanent colostomy) or LAR (anterior resection, involving the removal of the sigmoid colon and rectum to a level free of cancer, with a primary anastomosis between the descending colon and rectum or anal sphincter, thus avoiding the need for a permanent colostomy, and this procedure is preferred whenever possible).

To assess the association between treatment response and the type of treatment (TNT or NT), we employed the Chi-Square Test for Independence. Data analysis was performed using STATA MP 64; p-values less than 0.05 were considered statistically significant. All statistical tests were two-tailed.

## Results

We included 77 patients in our study analysis. Of these, 60 patients received standard (neoadjuvant treatment/NT) neoadjuvant chemoradiation, and 17 patients received treatment per the total neoadjuvant (TNT) approach. Most patients were of age > 65 and had an ECOG performance status of 0–1. There was a male predominance in the entire cohort, comprising 62% of our study population.

**Fig. 1:**
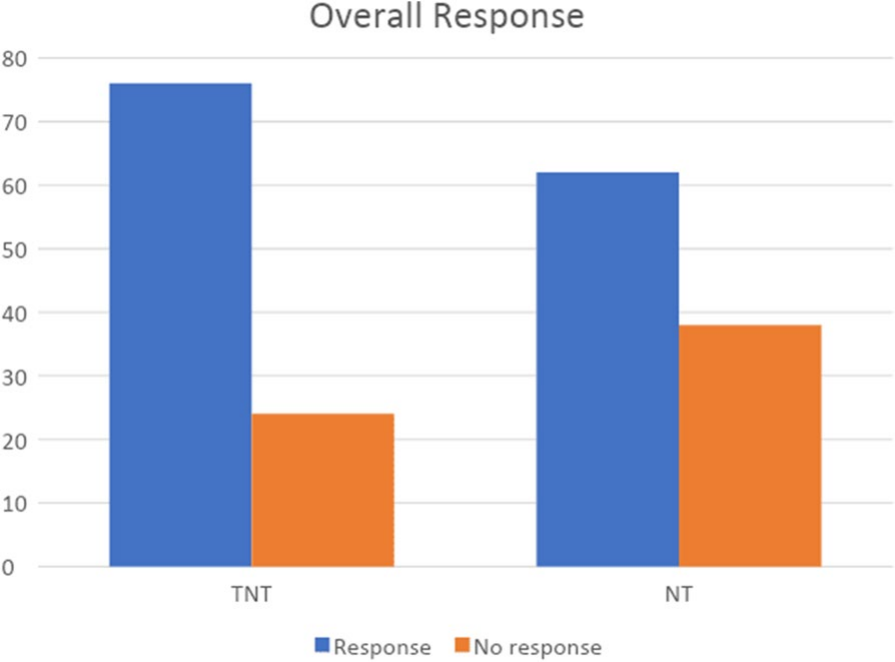
Response in TNT vs NT group

**Fig. 2:**
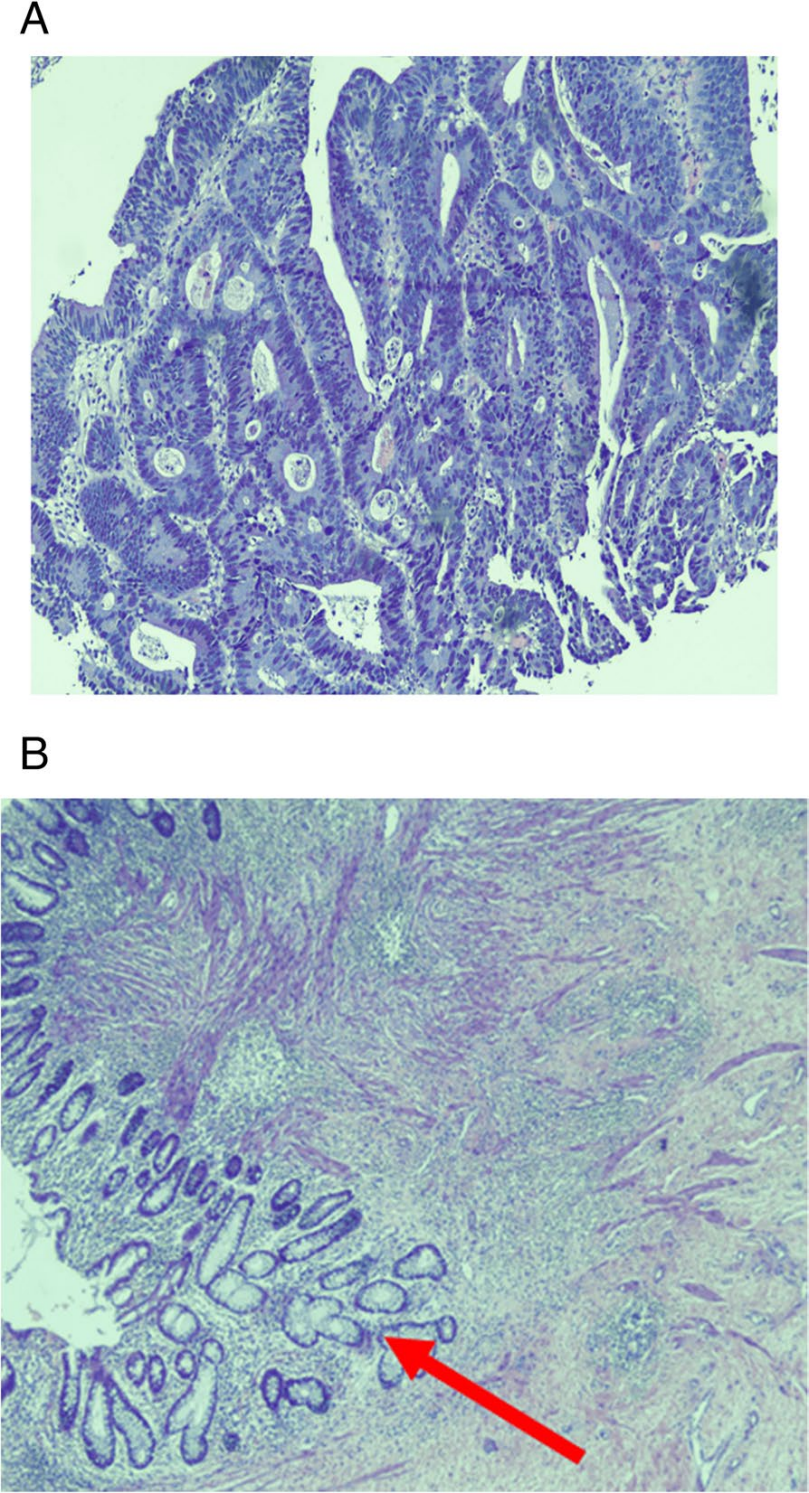
**A** Rectal Carcinoma forming glands exhibiting nuclear atypia and stratification. **B** Tumor bed area with fibrosis and inflammation (pCR) (Arrow: Normal rectal mucosa)

The overall pathological Response was assessed. Patients in the TNT group had statistically significant response rates compared to the NT group. (76% vs 62%, *p* = 0.039) (Fig. 1) (Table 4). Among the patients who responded to the treatment, 21 (40.38%) had complete pathological Response. (Fig. 2) represents the change in histopathology from invasive rectal adenocarcinoma on initial diagnosis to complete pathological Response after neoadjuvant therapy.

**Table 4:** Response to treatment

Response to treatment	Total Neoadjuvant Treatment (TNT) (n)	Neoadjuvant treatment (NT) (n)	Total (n)
Response	15	37	52
No response	02	23	25
Total	17	60	77

**Fig. 3:**
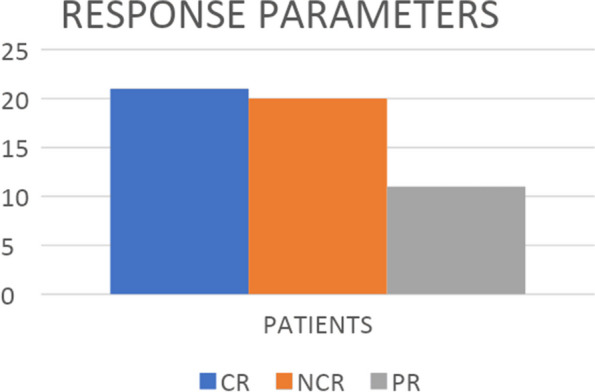
Complete Response (CR), Near Complete Response (Near-CR), and Partial Response(PR) among patients with pathological Response after treatment in the overall population

Among the total responders, there were almost equal number of patients with complete and near complete pathological responses, 27.2% VS 26% respectively, with partial response observed in 14% of the overall population (Fig. 3) (Table 5). The majority of the patients in both NT and TNT groups were clinical stage III rectal cancers (85% vs 100%). Surgical outcomes revealed that most patients in NT and TNT groups had T0-T2 (66.6% VS 70.58%) and N0-N1 (75% VS 88%) disease. Circumferential resection margin (CRM) was reviewed in all patients, and it was negative in 72% and 82% in the NT and TNT groups, respectively. Most of our patients received CAPEOX systemic chemotherapy in perioperative settings in both groups according to physician choice. There was no preference for surgical procedures.

**Table 5:** Types of pathological response

Treatment type	Pathological response			
	**Complete Response (pCR) (n)**	**Near Complete Response (Near CR) (n)**	**Partial Response (PR) (n)**	**Total (n)**
**Total Neoadjuvant treatment (TNT)**	09	02	04	15
**Neoadjuvant treatment (NT)**	12	09	16	37
**Total (n)**	21	20	11	52

**Fig. 4:**
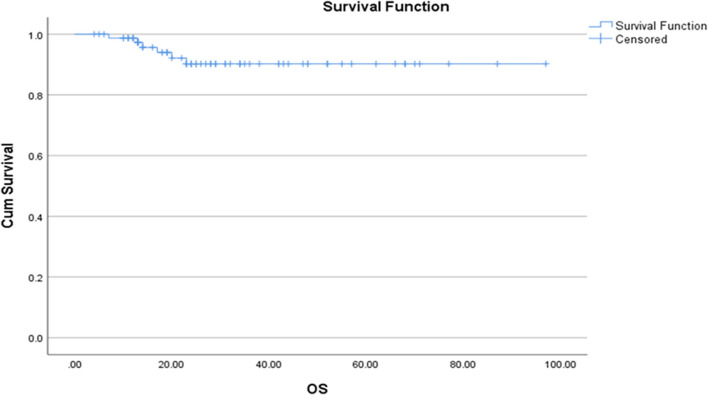
Mean overall survival

In the TNT group, a very limited number of patients had received a short course of radiation therapy. Out of 9 patients who had received short-course radiation, pCR was achieved in 6 patients. Perineural invasion (PNI) was seen in 16.66% and 17.64%, and lymphovascular invasion (LVI) was seen in 13.33% and 11.76% in NT and TNT groups, respectively. There was a significant association of treatment characteristics with the type of chemotherapy, concurrent chemoradiation therapy, LVI, PNI, and pathological Tumor (T), Nodal (N) and M (metastasis) staging in both the groups (*p* < 0.05). The most common side effects (of any grade) that were observed in the NT vs TNT group include neuropathy (25% vs 23.5%), diarrhea (6.6% vs 5.8%), oral mucositis (3.3% vs 17.6%) and thrombocytopenia (5% vs 0) (Table 6). Dose modification was done in 4 patients due to intolerance and acceptable side effects. The median overall survival was not reached, and the mean survival of the patient was 89.1 months (CI: 95.0 to 83.3) (Fig. 4).

**Table 6:** Side effects observed

Side effects observed	NT n (%)	TNTn (%)
Deranged liver function tests	01 (1.6)	01 (5.8)
Oral mucositis	02 (3.3)	03(17.6)
Diarrhea	04 (6.6)	01 (5.8)
Neuropathy	15 (25)	04 (23.5)
Thrombocytopenia	03 (5)	0
Skin manifestations	01 (1.6)	01(5.8)
Urinary tract infection	02(3.3)	01 (5.8)
Sexual problem	Not known	Not Known
None	13 (21.6)	05 (29.4)

## Discussion

The landscape of rectal cancer treatment has undergone a substantial transformation, shifting from traditional upfront surgery to the incorporation of radiation therapy and chemotherapy as part of concurrent chemoradiation treatment. This evolution underscores the importance of a multimodal approach in managing rectal cancer. The continuing adoption of neoadjuvant strategies, including standard chemoradiation to total neoadjuvant therapy (TNT), has become the standard of care [9]. Furthermore, with advancements in molecular genomics and next-generation sequencing analysis, treatment for rectal cancer is still evolving with the recent addition of immunotherapy in neoadjuvant settings in deficient mismatch repair (MMR) tumors.

Our study aligns with this paradigm shift, emphasizing the significance of neoadjuvant approaches in the management of rectal cancer within the Pakistani population. Neoadjuvant treatment, especially when chemotherapy is combined with radiation, has demonstrated superiority in enhancing local control and increasing the rate of pathological complete Response (pCR) compared to surgery alone [10, 11]. Our findings echo this trend, with a noteworthy % pCR rate of 27% in our real-world patient cohort falling within the range reported in clinical trials (pCR 8–28%) [12]. Pooled analyses highlight the importance of pCR as a primary outcome, correlating it with improved disease-free survival (DFS) in rectal cancer patients [13].

The neoadjuvant approach can be further categorized into total neoadjuvant therapy (TNT), which includes concurrent chemoradiation (CCRT) and 5-Fluorouracil (5-FU)-based chemotherapy followed by surgery. This approach has shown comparable outcomes to the conventional sequence of CCRT followed by surgery and chemotherapy, with the added advantage of better tolerance and increased completion rates of planned chemotherapy [14]. Our study underscores the practical implications of induction chemotherapy in facilitating treatment completion, providing valuable insights into real-world settings.

Various randomized clinical trials have explored the choice between long-course and short-course radiation in neoadjuvant settings. The Rapido trial in 2021 reported lower 3-year disease-related treatment failure with short-course radiotherapy compared to the standard therapy group [15]. In our study, a limited number of patients who received short-course radiation therapy exhibited a promising pCR rate. However, the small sample size prevented a comprehensive evaluation of this subgroup. Future studies with a larger patient population are essential to delineate the outcomes of this emerging approach.

An intriguing development in rectal cancer management is the emergence of the "wait and watch" strategy, particularly in patients with clinically complete responses. The OPRA trial demonstrated similar disease-free survival at three years, suggesting that a conservative approach may be suitable for selected patients [16]. In our study, all patients underwent a surgical approach, and adopting a "watch and wait" strategy was not explored. Incorporating this approach in future studies could provide valuable insights into its applicability and outcomes in our population.

The molecular landscape of rectal cancer is evolving, with microsatellite-high (MSI-high) status guiding the choice of neoadjuvant immunotherapy. Currently, pembrolizumab or dostarlimab for six months in the neoadjuvant setting is preferred for locally advanced rectal cancer patients with MSI-high status [6, 8, 17, 18]. Our study did not include information on MSI status; none of the patients underwent immunotherapy in the neoadjuvant setting. Future investigations should consider incorporating molecular testing and evaluating the role of immunotherapy in our population.

Circumferential resection margin (CRM) status plays a crucial prognostic role in locally advanced rectal cancer, as documented in various studies within the literature. Patients with a positive CRM often exhibit heightened early local recurrence and distant relapse rates, emphasizing the need for thorough investigation and management strategies [12, 19]. Our comprehensive study analyzed the CRM status in neoadjuvant therapy (NT) and total neoadjuvant therapy (TNT) groups. Remarkably, less than 10% of patients in both groups displayed a positive CRM, aligning with existing literature trends. This finding underscores the importance of effective therapeutic interventions to minimize the occurrence of positive CRM in rectal cancer patients. Beyond CRM, our study delved into the prognostic significance of lymphovascular invasion (LVI) and perineural invasion (PNI). The literature reports LVI positivity rates ranging from 15–30% and PNI positivity rates from 10–20% [6, 19, 20, 21, 22]. Encouragingly, our study corroborated these figures in both the NT and TNT groups, reinforcing the consistent prognostic relevance of these invasion markers. Recognizing the dynamic nature of cancer prognosis, our study advocates for long-term follow-up of these patients to monitor early recurrence patterns.

Despite its strengths, our study has limitations. Our investigation entailed a retrospective analysis focusing on Stage II and III rectal cancers exclusively within our institution. Data collection spanned from January 2017 to December 2022. Among the 800 patients assessed, the predominant portion consisted of individuals with metastatic disease upon diagnosis, those who underwent upfront resection, or those who did not meet eligibility criteria. Given the publication of TNT trials such as Rapido, Prodige-23, and Stellar between 2020 and 2021[15, 16, 21], there was a transitional period before the adoption of the total neoadjuvant approach, resulting in the majority of our patients still receiving the neoadjuvant approach. Furthermore, the limitation of a small sample size is indeed a significant one. The high APR rate observed in our study may not be representative of a larger population due to the small number of patients who underwent the TNT approach (17 patients), of which 11 required APR. Similar to this, in stellar trial in TNT group around 45% of the patient underwent APR [21, 23]. We recognize this and suggest that our findings should be interpreted with caution. It is plausible that in a larger, more heterogeneous cohort, the percentage of patients requiring APR might different. Additionally, median overall survival data is pending, and continued follow-up is essential to determine the survival benefit associated with pCR. In our study, toxicity analysis could not be recorded according to the Common Terminology Criteria for Adverse Events (CTCAE) as this was a retrospective review and medical records do not document every toxicity according to grades in CTCAE. The small number of patients in the TNT and short-course radiation groups underscores the need for larger studies to validate our findings and comprehensively explore the outcomes in these subpopulations.

## Conclusion

Our study contributes to the growing body of evidence supporting the effectiveness of neoadjuvant and total neoadjuvant approaches in the management of rectal cancer in the Pakistani population. While the study demonstrates a favourable pCR rate, further research with extended follow-up is imperative to ascertain the overall survival benefit and validate the outcomes in specific subgroups, such as those receiving short-course radiation therapy. The dynamic landscape of rectal cancer treatment warrants ongoing exploration and adaptation of therapeutic strategies for improved patient outcomes.

## Data Availability

The data that has been used is confidential. The datasets are available from the corresponding author.

## Abbreviations

ECOG: European cooperative oncology group
TNT: Total neoadjuvant treatment
NT: Neoadjuvant treatment
PCR: Pathological complete response
PR: Partial response
LVI: Lymphovascular space invasion
PNI: Perineural invasion
